# *pts* promoter influences antibiotic resistance via proton motive force and ROS in *Escherichia coli*

**DOI:** 10.3389/fmicb.2023.1276954

**Published:** 2023-11-06

**Authors:** Jian-jun Tao, Shao-hua Li, Jia-han Wu, Xuan-xian Peng, Hui Li

**Affiliations:** ^1^State Key Laboratory of Bio-Control, Southern Marine Science and Engineering Guangdong Laboratory (Zhuhai), Guangdong Province Key Laboratory for Pharmaceutical Functional Genes, School of Life Sciences, Sun Yat-sen University, Guangzhou, China; ^2^Laboratory for Marine Fisheries Science and Food Production Processes, Qingdao National Laboratory for Marine Science and Technology, Qingdao, China; ^3^Guangdong Litai Pharmaceutical Co. LTD, Jieyang, China

**Keywords:** *Escherichia coli*, PTS, glucose, antibiotic resistance, reprogramming metabolomics, the pyruvate cycle

## Abstract

**Introduction:**

Glucose level is related to antibiotic resistance. However, underlying mechanisms are largely unknown.

**Methods:**

Since glucose transport is performed by phosphotransferase system (PTS) in bacteria, *pts* promoter-deleted K12 (Δ*pts*-P) was used as a model to investigate effect of glucose metabolism on antibiotic resistance. Gas chromatography-mass spectrometry based metabolomics was employed to identify a differential metabolome in Δ*pts*-P compared with K12, and with glucose as controls.

**Results:**

Δ*pts*-P exhibits the resistance to β-lactams and aminoglycosides but not to quinolones, tetracyclines, and macrolide antibiotics. Inactivated pyruvate cycle was determined as the most characteristic feature in Δ*pts*-P, which may influence proton motive force (PMF), reactive oxygen species (ROS), and nitric oxide (NO) that are related to antibiotic resistance. Thus, they were regarded as three ways for the following study. Glucose promoted PMF and β-lactams-, aminoglycosides-, quinolones-mediated killing in K12, which was inhibited by carbonyl cyanide 3-chlorophenylhydrazone. Exogenous glucose did not elevated ROS in K12 and Δ*pts*-P, but the loss of *pts* promoter reduced ROS by approximately 1/5, which was related to antibiotic resistance. However, NO was neither changed nor related to antibiotic resistance.

**Discussion:**

These results reveal that *pts* promoter regulation confers antibiotic resistance via PMF and ROS in *Escherichia coli*.

## Introduction

The rise and dissemination of antibiotic resistance due to the widespread and inappropriate utilization of antibiotics pose a significant and imminent risk to human health, medical practices, and societal well-being (Nadimpalli et al., 2020). Recently, Antimicrobial Resistance Collaboration has provided a comprehensive global estimate of the impact of antimicrobial resistance, which is associated with 4.95 million deaths in 2019 (Laxminarayan, 2022). The situation is projected to worsen, with an estimated 10 million deaths from drug-resistant bacterial infections anticipated by 2050 (Merker et al., 2020). Therefore, a deeper understanding of antibiotic resistance mechanisms is of paramount importance and urgency to devise innovative therapeutic approaches against antibiotic-resistant pathogens (Peng et al., 2019, 2023).

A line of evidence has indicated that bacterial metabolism contributes to antibiotic resistance/susceptibility, from which crucial metabolites are identified to potentiate antibiotics to kill antibiotic-resistant bacteria (Allison et al., 2011; Peng et al., 2015a; Su et al., 2018; Cheng et al., 2019; Yang et al., 2023). This is because antibiotic-resistant and -sensitive bacteria have antibiotic-resistant and -sensitive metabolic states, respectively. Crucial biomarkers identified from the comparison between antibiotic-resistant and -sensitive metabolic states can be used as metabolic state-reprogramming metabolites to reprogram antibiotic-resistant metabolic states into antibiotic-sensitive metabolic states (Peng et al., 2015b; Li et al., 2020; Kuang et al., 2021; Tang et al., 2022; Yin et al., 2022; Chen et al., 2023; Yang et al., 2023). For example, multidrug-resistant *Escherichia coli* have a specific metabolic state with inactivated alanine, aspartate, and glutamate metabolism and repressed glutamine as the most metabolic characteristic feature of multidrug resistance, which causes the bacteria to take up fewer antibiotics (Zhao et al., 2021). Exogenous glutamine reverses the resistant metabolic state into a sensitive metabolic state and thereby allows antibiotic-resistant bacteria to take up more antibiotics for rapidly elevating intracellular drug concentration, leading to high killing efficacy regarding antibiotic-resistant bacteria (Zhao et al., 2021; Chen et al., 2022). Therefore, the impairment of key biomarker-mediated metabolism will affect antibiotic sensitivity.

Several reports have shown that the depression of glucose is the most characteristic consequence of bacterial antibiotic resistance (Peng et al., 2015b; Zhang et al., 2019, 2020; Tang et al., 2022). Exogenous glucose as a metabolic state-reprogramming metabolite effectively potentiates aminoglycosides (kanamycin, gentamicin, and amikacin) to eliminate multidrug-resistant *Escherichia coli*, multidrug-resistant *Edwardsiella tarda*, cefoperazone/sulbactam-resistant *Pseudomonas aeruginosa*, and gentamicin-resistant *Vibrio alginolyticus* and their persisters and biofilms (Allison et al., 2011; Peng et al., 2015b; Zhang et al., 2019, 2020; Chen et al., 2022; Tang et al., 2022). Our recent dynamic observation of a metabolic state has shown that glucose abundance decreases progressively as ampicillin-sensitive strains acquire resistance to ampicillin. This is linked to *pts* promoter, which controls glucose transport. Deletion of *pts* promoter in clinically isolated antibiotic-sensitive *E. coli* strains confers ampicillin resistance even in the presence of glucose (Jiang et al., 2023). These data indicate that the reduced intracellular glucose due to the absence of *pts* promoter promotes bacterial resistance to ampicillin. *Pts* promoter controls the expression of the phosphotransferase system (PTS), which manages the uptake and phosphorylation of glucose (Hernández-Montalvo et al., 2003). Therefore, *pts* promoter-deleted cells are an ideal model that explores glucose-controlled antibiotic resistance.

β-lactams, aminoglycosides, and quinolones are the three most commonly used antibiotic classes in the clinic setting. In addition, tetracyclines and macrolides are important supplements for the clinically used antibiotic classes. β-lactams primarily target the penicillin-binding proteins (PBPs) to render PBPs unavailable for the synthesis of new peptidoglycan. This disruption in the peptidoglycan layer ultimately leads to bacterial lysis (Kapoor et al., 2017). Aminoglycosides form hydrogen bonds with the 16S rRNA of the 30S subunit near the A site, leading to mRNA misreading and premature translation termination (Kapoor et al., 2017). Quinolones work by inhibiting the bacterial enzyme DNA gyrase, which is responsible for introducing negative supercoils into double-stranded DNA to prevent the enzyme from resealing the nicked ends of the DNA (Higgins et al., 2003). Tetracyclines act on the conserved sequences of the 16S rRNA of the 30S ribosomal subunit to inhibit tRNA binding at the A site (Yoneyama and Katsumata, 2006). Macrolides target the conserved sequences of the peptidyl transferase center of the 23S rRNA of the 50S ribosomal subunit, primarily affecting the early stage of protein synthesis, known as translocation (Yoneyama and Katsumata, 2006). These antibiotics play an important role in saving patients’ lives and safeguarding people’s health.

Here, *pts* promoter was genetically deleted in model *E. coli* K12 BW25113 (Δ*pts-*P) to explore antibiotic resistance mechanisms mediated by depressed glucose. For this purpose, besides K12 and Δ*pts-*P, two additional groups, K12 + glucose and Δ*pts-*P + glucose, were included as positive and negative controls, respectively. This is because the two strains of bacteria respond differently to glucose, which provides metabolites that change in the opposite direction for facilitating the identification of glucose-regulated metabolites. Gas chromatography–mass spectrometry (GC–MS) based metabolomics with biochemistry analysis showed that the pyruvate cycle (the P cycle) is depressed, which is the most affected metabolic pathway. This depression downregulated proton motive force (PMF). Meanwhile, the loss of *pts* promoter reduced ROS. They made bacteria resistant to β-lactams and aminoglycosides.

## Results

### Resistance phenotypes of Δ*pts-*P

**Figure 1:**
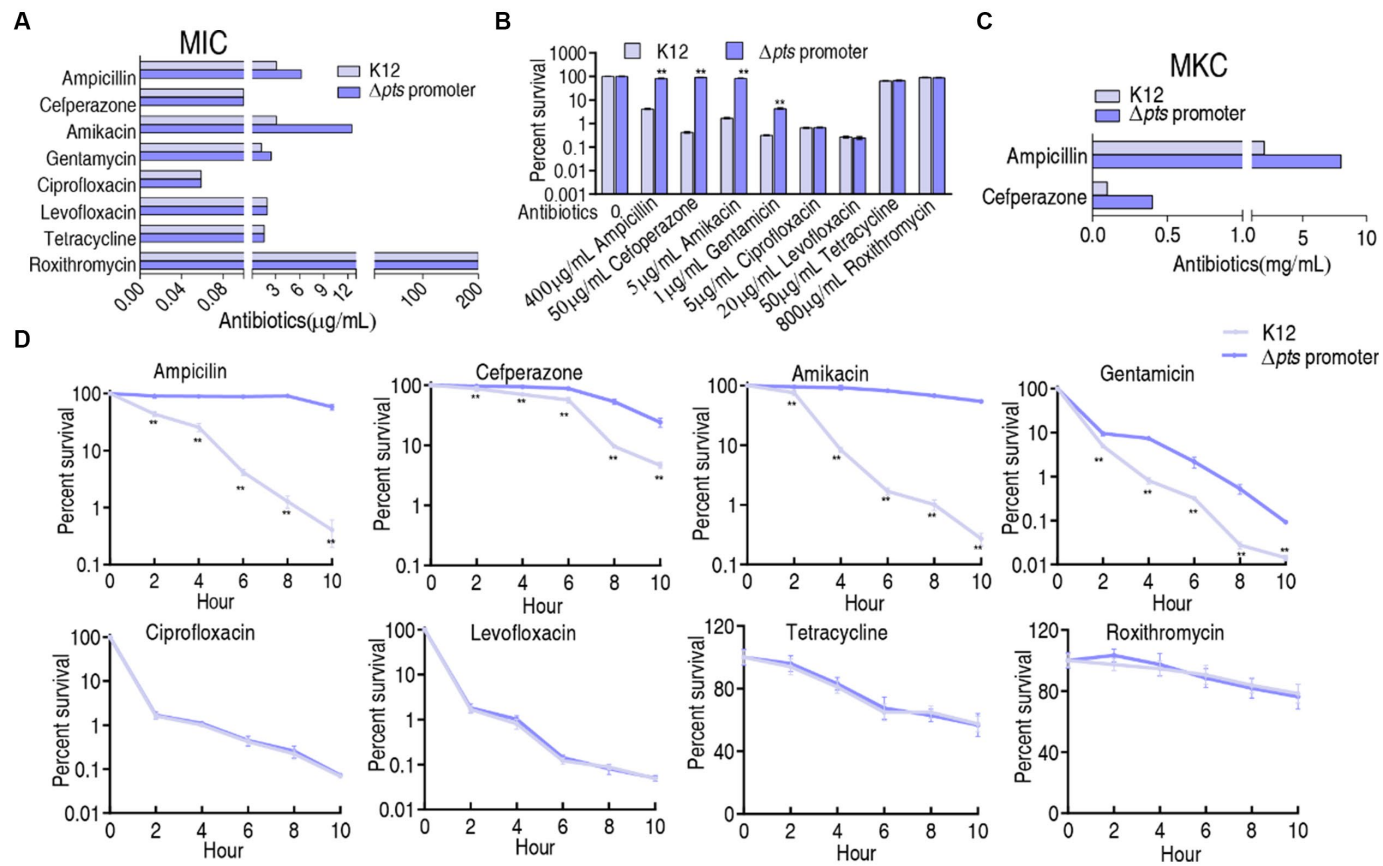
Resistance phenotypes in K12 and Δ*pts*-P. **(A)** Minimum inhibitory concentration (MIC) of the two strains to each of the eight antibiotics. **(B)** Survival of the two strains in the presence of the indicated concentrations of antibiotics. **(C)** Minimum killing concentration (MKC) of the two strains to ampicillin and cefperazone. **(D)** Survival of the two strains in the presence of the indicated incubation time plus antibiotics as the same as in data **(B)**. Results are displayed as mean ± SEM and three biological repeats are performed. Significant differences are identified **p < 0.01.

K12 and Δ*pts*-P strains were cultured in LB medium with or without each of the eight antibiotics for the measurement of minimum inhibitory concentration (MIC). The MIC for each antibiotic tested was consistent among three biological replicates of the two strains. For the K12 strain, 3.125 μg/mL ampicillin and 0.1 μg/mL cefperazone (β-lactams), 3.125 μg/mL amikacin and 1.25 μg/mL gentamycin (aminoglycosides), 0.022 μg/mL ciprofloxacin and 0.059 μg/mL levofloxacin (qunolones), and 1.98 μg/mL tetracycline (tetracyclines) and 200 μg/mL roxithromycin (macrolides) were defined as 1 MIC. However, the absence of *pts* promoter caused increased MIC at amikacin (12.5 μg/mL, 4 MIC), ampicillin (6.25 μg/mL, 2 MIC), and gentamycin (2.5 μg/mL, 2 MIC), and maintained unchanged MIC at the other five antibiotics (Figure 1A). Furthermore, a survival capability test was performed, which is more sensitive than MIC measurement (Li et al., 2007). Survival of the two strains was measured in M9 medium with each of the eight antibiotics. Higher survival was detected in the mutant than in K12 in the presence β-lactams (ampicillin and cefperazone) and aminoglycosides (amikacin and gentamycin) antibiotics but not in the complementation of quinolones (ciprofloxacin and levofloxacin), tetracyclines (tetracycline), and macrolides (roxithromycin) (Figure 1B). In M9 medium, some antibiotics require the use of high concentrations, for example, ampicillin (128-fold MIC) and cefoperazone (500-fold MIC) were used. This is because bacterial antibiotic resistance is increased in M9 medium. However, bacteria do not grow in M9 medium and, thereby, bacterial resistance to β-lactams is measured using minimum killing concentration (MKC) instead of MIC (Tao et al., 2022). Indeed, higher drug concentration was measured in MKC than in MIC (Figure 1C). Furthermore, a time-kill assay was performed to confirm the killing. Viability of K12 and Δ*pts*-P was reduced with increasing incubation periods in the eight drugs. However, lower viability was detected in K12 than Δ*pts*-P when ampicillin, cefperazone, amikacin, or gentamycin was used, but it remained stable when these drugs were replaced with quinolones (ciprofloxacin or levofloxacin), tetracyclines (tetracycline), and macrolides (roxithromycin) (Figure 1D). These results indicate that the loss of *pts* promoter exhibits increased resistance to β-lactams and aminoglycosides but not quinolones, tetracyclines, and macrolides, suggesting that *pts* regulation of antibiotic resistance is related to antibiotic classes. Note that Δ*pts*-P is still susceptible to β-lactams and aminoglycosides, based on the Clinical Laboratory Standards Institute and EUCAST standards (Kassim et al., 2016).

### Metabolic profiles of K12 and Δ*pts*-P with and without glucose

**Figure 2:**
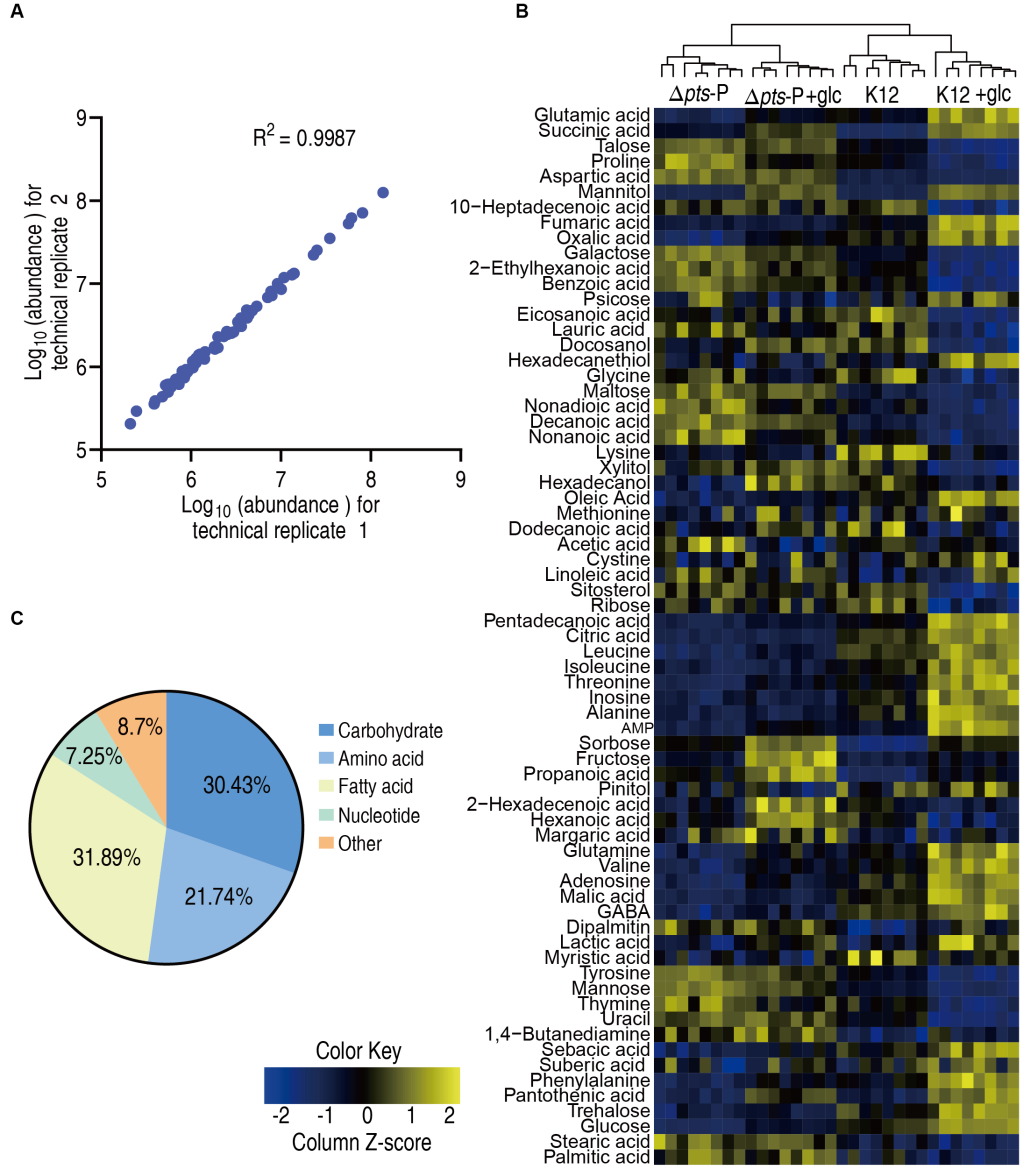
Metabolic profiles in K12 and Δ*pts*-P with and without glucose. **(A)** Correlation coefficient between technical replicates. **(B)** Heat map of unsupervised hierarchical clustering of all metabolites (row). Yellow and blue colors indicate increase and decrease of the metabolites scaled to mean and standard deviation of row metabolite level, respectively (see color scale). **(C)** Categories of all identified metabolites.

To explore the metabolic changes associated with the resistance, a metabolomics approach based on GC–MS was employed to compare the metabolic profiles of K12 and Δ*pts*-P with or without glucose, designated as K12, Δ*pts-*P, K12 + glc, and Δ*pts-*P + glc, respectively. A total of 32 data sets were obtained from four biological samples with two technical replicates for each group. The correlation coefficient between the technical replicates ranged from 0.995 to 0.999, indicating the high reproducibility of the data (Figure 2A). Metabolic profiles with 69 metabolites for each group were displayed as a heatmap, where the four groups were clustered independently and then K12 with K12 + glucose and Δ*pts*-P with Δ*pts-*P + glucose were separately grouped (Figure 2B). Based on KEGG annotation and NCBI PubChem, the 69 metabolites were classified into five categories, namely, carbohydrates (30.43%), amino acids (21.74%), fatty acids (31.89%), nucleotides (7.25%), and others (8.7%) (Figure 2C). These results indicate that these metabolites, especially carbohydrates, amino acids, fatty acids, and nucleotides, provide a metabolic basis for exploring *pts*-related antibiotic resistance.

### Differential metabolomes of K12 and Δ*pts*-P with and without glucose

**Figure 3:**
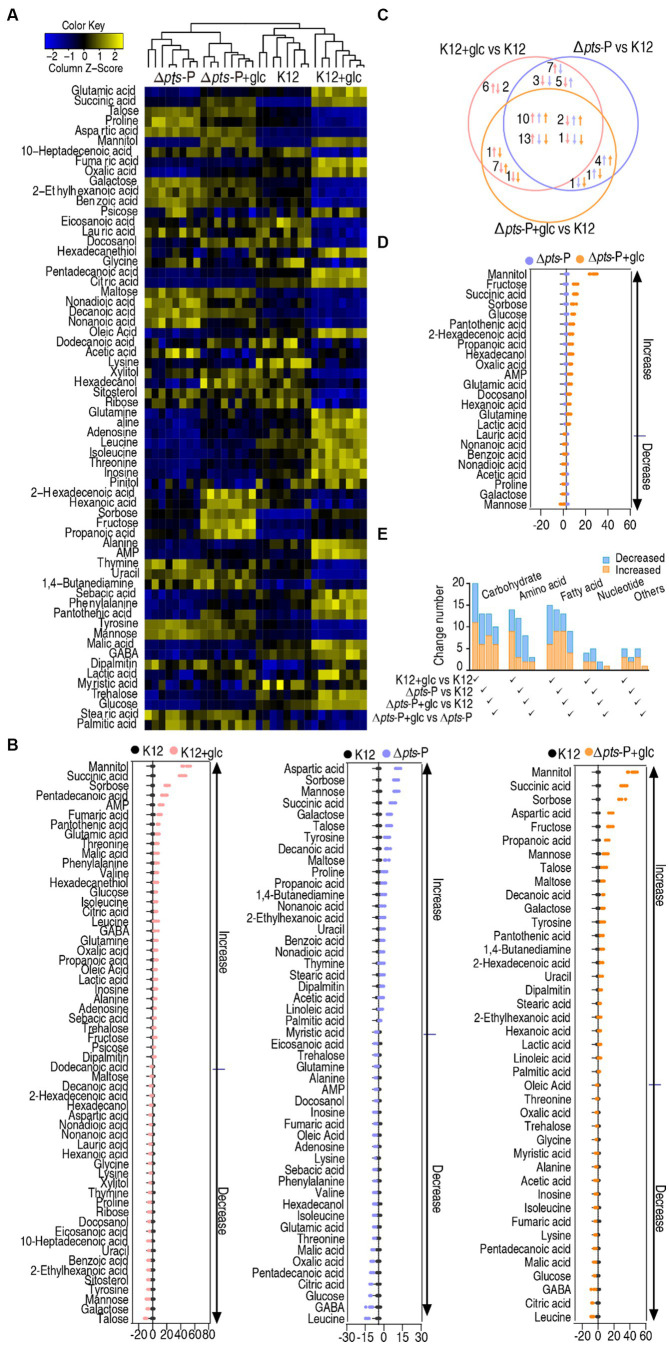
Glucose-induced differential metabolomics. **(A)** Heat map showing differential metabolites in four groups. Yellow and blue colors indicate increase and decrease of the metabolites scaled to mean and standard deviation of row metabolite level, respectively (see color scale). **(B)** *Z*-score plot of differential metabolites in data **(A)**. The data were separately scaled to the mean and SD of control K12. Each point represents one metabolite in one technical repeat and is colored by sample type. **(C)** Venn diagram for comparison of glucose-induced differential metabolites in K12 + glucose, Δ*pts*-P, and Δ*pts*-P + glucose based on control K12. Upward and downward arrows indicate increase and decrease, respectively, at metabolite abundance compared to the control group. **(D)** *Z*-score plot of differential metabolites in Δ*pts*-P + glucose compared with Δ*pts*-P. The data were separately scaled to the mean and SD of Δ*pts*-P. Each point represents one metabolite in one technical repeat and is colored by sample type. **(E)** Number and classification of differential abundance of metabolites based on KEGG annotation and NCBI PubChem.

A Kruskal-Wallis test (*p* < 0.01) was employed to identify differentially abundant metabolites. A total of 58, 47, and 41 differential metabolites were detected in K12 + glucose, Δ*pts*-P, and Δ*pts*-P + glucose, respectively, compared to K12 (Figure 3A). To display deviations between a value and the mean, a *Z*-score calculation was used. The *Z*-score measures exactly how many standard deviations above or below the mean a data point is. It varied between −10.4 and 47.5 in K12 + glucose, −10.8 and 18.4 in Δ*pts*-P, and − 7.3 and 42.6 in Δ*pts*-P + glucose, respectively. Among the 58, 47, and 41 differential metabolites, 37, 22, and 23 were upregulated and 21, 25, and 18 were downregulated, respectively. Particularly, metabolites (citric acid, fumaric acid, and malic acid) of the pyruvate cycle (the P cycle) were upregulated in the K12 + glucose group but downregulated in the Δ*pts-*P group and Δ*pts-*P + glucose group (Figure 3B). The Venn diagram illustrates the relationship of these differential metabolites among the three groups. We were interested in the metabolites that were increased in the K12 + glucose group but decreased in Δ*pts*-P and Δ*pts*-P + glucose or vice versa because they were related to glucose metabolism. In total, 13 metabolites were elevated in the K12 + glucose group but decreased in Δ*pts*-P and Δ*pts*-P + glucose, while 2 metabolites exhibited the opposite abundance (Figure 3C). On the other hand, a comparison was performed between Δ*pts*-P + glucose and Δ*pts*-P. A total of 24 differentially abundant metabolites that varied between −3.8 and 29.1 were detected in Δ*pts*-P + glucose. Among them, 16 were upregulated and 8 were downregulated (Figure 3D). All differential abundances of metabolites were categorized by KEGG annotation and NCBI PubChem and listed in (Figure 3E). These results suggest that these differential metabolites are manipulated by *pts* promoter via glucose. Therefore, exogenous glucose weakly changed the effect of the promoter absence on the metabolic profile. Since *pts* controls glucose transport, these results support the conclusion that *pts* plays a crucial role in the utilization of glucose.

### Metabolic pathway enrichment of K12 + glucose and Δ*pts*-P with and without glucose

**Figure 4:**
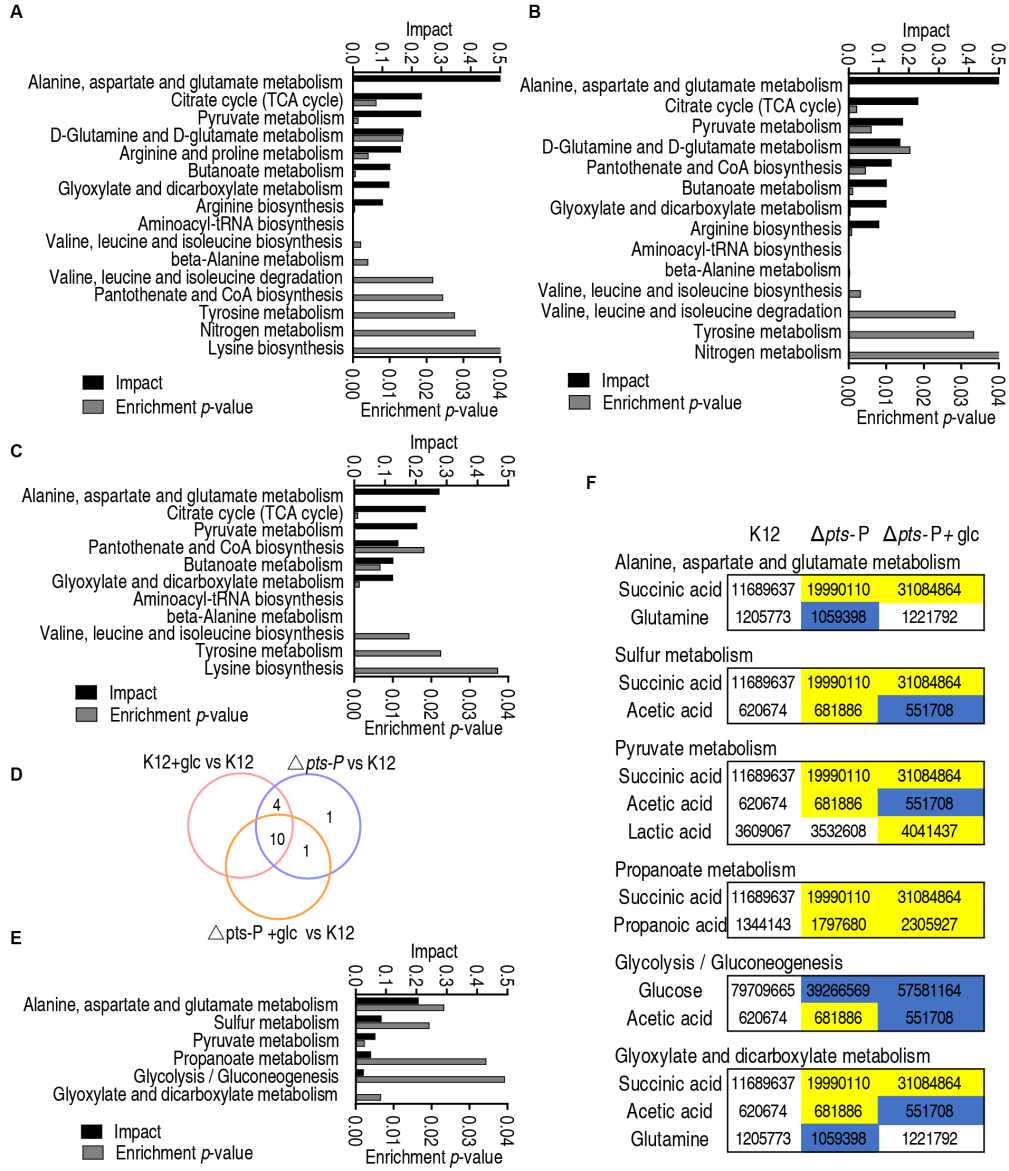
Pathway enrichment analysis. **(A–C)** Pathway enrichment in Δ*pts*-P **(A)**, K12 + glucose **(B)**, and Δ*pts*-P + glucose **(C)**, compared with K12. **(D)** Venn diagram for comparison in data **(A–C)**. **(E)** Pathway enrichment in Δ*pts*-P + glucose compared with Δ*pts*-P. **(F)** Integrative analysis of metabolites in significantly enriched pathways in data **(E)**.

Metabolic pathways represent a collection of biochemical reactions that are essential for cellular functions. Therefore, these enriched metabolic pathways provide valuable knowledge about the impact of *pts* promoter deletion on cellular metabolism. In total, 16, 14, and 11 metabolic pathways were enriched in Δ*pts*-P, K12 + glucose, and Δ*pts*-P + glucose, respectively, based on the differential metabolites compared to K12. (Figures 4A–C). Similar numbers of metabolic pathways were enriched in both Δ*pts*-P and K12 + glucose, which were higher than those of Δ*pts*-P + glucose. Among these pathways, 10 were overlapped in the three groups, 4 were shared by Δ*pts*-P and K12 + glucose, 1 was shared by Δ*pts*-P + glucose and Δ*pts*-P, and 1 was specific for Δ*pts*-P (Figure 4D). Interestingly, exogenous glucose caused Δ*pts*-P to be far from K12 + glucose. Among the 10 overlapped metabolic pathways, the top 3 ranked pathways (alanine, aspartate, and glutamate metabolism; the TCA cycle/ P cycle; and pyruvate metabolism) are included. On the other hand, 6 metabolic pathways were enriched by differential metabolites in Δ*pts*-P + glucose compared with Δ*pts*-P. Among them, 3 (alanine, aspartate, and glutamate metabolism; pyruvate metabolism; and glyoxylate and dicarboxylate metabolism) were included in the above 10 overlapped metabolic pathways, while 3 (sulfur metabolism, propanoate metabolism, and glycolysis/gluconeogenesis) were specific for the enrichment (Figure 4E). Specifically, succinic acid, glutamine, acetic acid, lactic acid, propanoic acid, and glucose were responsible for the enrichment. Taking K12 as a control, high succinic acid, propanoic acid, and low glucose were detected in Δ*pts*-P and Δ*pts*-P + glucose, where succinic acid, propanoic acid and glucose were lower in Δ*pts*-P than Δ*pts*-P + glucose; lower glutamine was measured in Δ*pts*-P but stable in Δ*pts*-P + glucose; higher lactic acid was determined in Δ*pts*-P + glucose but stable in Δ*pts*-P; higher and lower acetic acid was found in Δ*pts*-P and Δ*pts*-P + glucose, respectively (Figure 4F). Meanwhile, the TCA cycle/ P cycle was not enriched. These results suggest that exogenous glucose weakly affects the metabolic pathways due to the absence of *pts* promoter. Therefore, the TCA cycle/ P cycle is regarded as the key metabolic pathway affected by glucose.

### *pts*-manipulated metabolites

**Figure 5:**
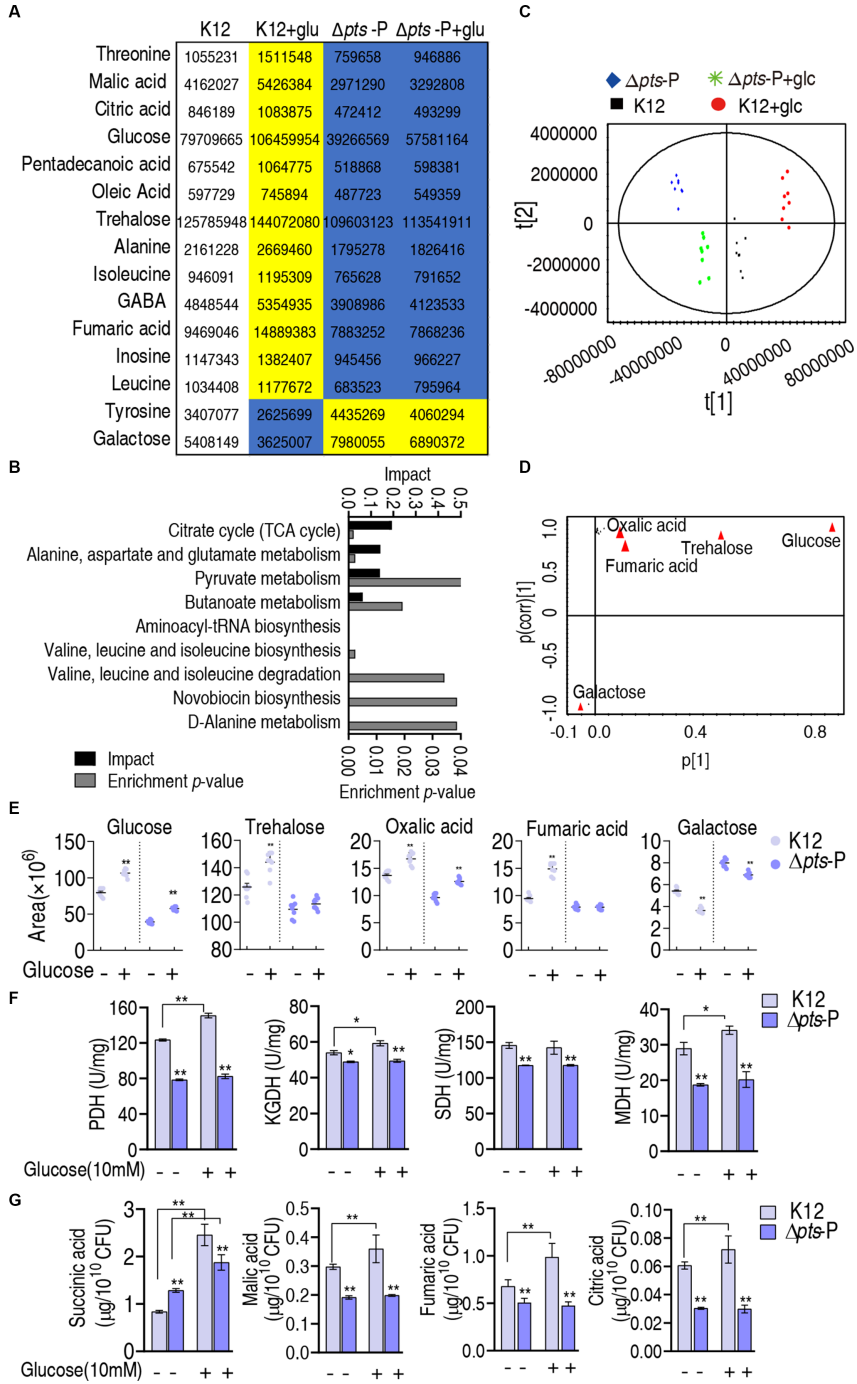
Identification of crucial pathways and biomarkers. **(A)** Abundance of key metabolites i K12 + glucose, Δ*pts*-P, and Δ*pts*-P + glucose, compared with control K12. Yellow and blue colors indicate increased and decreased metabolites, respectively. **(B)** Pathway enrichment of key metabolites. **(C)** Principal component analysis (PCA) of the four strains. Each dot represents the technical replicate of samples in the plot. **(D)** The S-plot generated from OPLS-DA illustrates individual metabolites as triangles. Potential biomarkers, indicated in red, are those with an absolute value of covariance *p* greater than or equal to 0.05 and correlation *p* (corr) greater than or equal to 0.5. **(E)** The scatter plot depicting the biomarkers in data. **(F)** Activity of PDH, KGDH, SDH, and MDH in the P cycle in the presence or absence of glucose. **(G)** Quantification of succinic acid, malic acid, fumaric acid, and citric acid in K12 and Δ*pts*-P with and without glucose. Results are displayed as mean ± SEM and three biological repeats are performed. Significant differences are identified *p < 0.05, **p < 0.01.

Further analysis was focused on the differential abundances of metabolites between K12 + glucose and Δ*pts*-P with and without glucose because they represented these metabolites that are influenced due to the loss of *pts* promoter. A total of 15 metabolites were identified, where 13 were downregulated and 2 were upregulated (Figure 5A). They led to the enrichment of 9 metabolic pathways. According to the impact, they were ranked from high to low as follows: the TCA cycle/ P cycle > alanine, aspartate, and glutamate metabolism > pyruvate metabolism > butanoate metabolism > aminoacyl-tRNA biosynthesis > valine, leucine, isoleucine biosynthesis > valine, leucine, isoleucine degradation > novobiocin biosynthesis > D-alanine metabolism (Figure 5B). Then, orthogonal partial least square-discriminate analysis (OPLS-DA) was conducted to recognize the sample pattern to obtain component t [1] and component t [2]. Component t [1] differentiated K12 and K12 + glucose from Δ*pts-*P and Δ*pts*-P + glucose, while component t [2] separated Δ*pts*-P + glucose and K12 from Δ*pts*-P and K12 + glucose (Figure 5C). An S-plot was generated to visualize the discriminating variables. Out of these metabolites, five were identified as biomarkers due to their values ≥0.05 and ≥0.5 for the absolute value of covariance p and correlation p (corr), respectively. These biomarkers play a more significant role in the differentiation process, where glucose exhibited the highest weight (Figure 5D). The scatter plot shows their differential abundances in K12 + glucose, Δ*pts*-P, and Δ*pts*-P + glucose compared with those in K12. Specifically, among the five biomarkers, level of glucose, trehalose, oxalic acid, and fumaric acid was ranked from high to low as K12 + glucose > K12 > Δ*pts*-P + glucose and Δ*pts*-P (level of glucose and oxalic acid was higher in Δ*pts*-P + glucose than Δ*pts*-P), while the level of galactose was higher in Δ*pts*-P and Δ*pts*-P + glucose than in K12 and K12 + glucose (Figure 5E), suggesting that glucose metabolism influences galactose product. Since glucose flux enters the P cycle, and biomarker (reduced fumaric acid) and differential metabolites (reduced citric acid and malic acid and elevated succinic acid) are intermetabolites of the P cycle (Su et al., 2018), the P cycle was identified as the most affected metabolic pathway. Therefore, the activity of pyruvate dehydrogenase (PDH), α-ketoglutarate dehydrogenase (KGDH), succinate dehydrogenase (SDH), and malate dehydrogenase (MDH) in the P cycle was measured to support the conclusion. Compared to K12, the activity of these four enzymes was reduced in both Δ*pts*-P and Δ*pts*-P + glucose. However, exogenous glucose supplementation increased the activity of PDH, KGDH, and MDH in K12 but did not influence the activity of the four enzymes in Δ*pts*-P (Figure 5F). Using GC–MS, a higher abundance of malic acid, fumaric acid, and citric acid and a lower abundance of succinic acid were measured in K12 than in Δ*pts*-P. When exogenous glucose was added, no changes in malic acid, fumaric acid, and citric acid but a high abundance of succinic acid were detected in Δ*pts*-P compared with K12 (Figure 5G). Speculatively, the elevated succinic acid of the P cycle is related to the activated glyoxylic acid shunt in antibiotic-resistant bacteria (Bhusal et al., 2017; Zhong et al., 2020) and the enzyme activity changed disproportionately, which requires further investigation. These results indicate that the loss of *pts* promoter inhibits the glucose flux to the P cycle, leading to the inactivation of the P cycle.

### Effect on proton motive force, ROS, and NO in K12 and Δ*pts-*P with and without glucose

**Figure 6:**
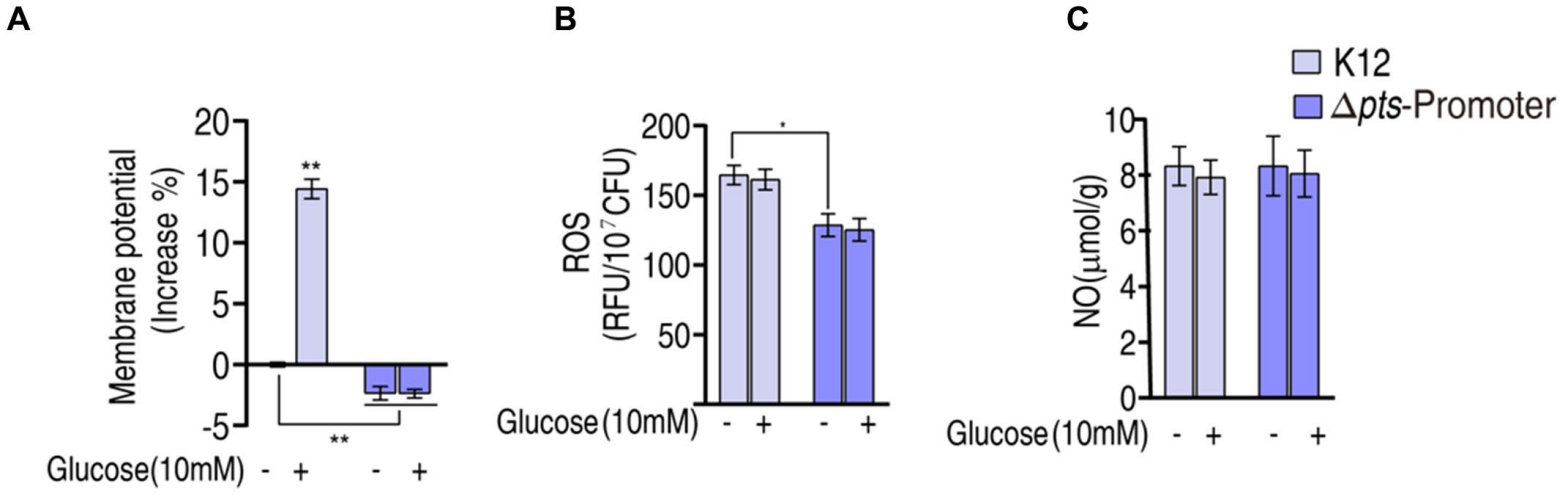
Potential mechanisms. **(A)** Membrane potential measurement. **(B)** ROS measurement in K12 and Δ*pts*-P with and without glucose. **(C)** NO measurement in K12 and Δ*pts*-P in K12 and Δ*pts*-P with and without glucose. Results are displayed as mean ± SEM and three biological repeats are performed. Significant differences are identified *p < 0.05, **p < 0.01.

The above results show that *pts* controls glucose transport, which affects the P cycle. Reports have indicated that the inactivated P cycle is linked to antibiotic resistance via proton motive force (PMF), ROS, and nitric oxide (NO) (Zhang et al., 2019, 2020; Kuang et al., 2021; Ye et al., 2021; Zhao et al., 2021; Mao et al., 2022). Of them, PMF provides a force to promote drug uptake, and ROS and NO are related to antibiotic susceptibility (Peng et al., 2015b; Kuang et al., 2021; Ye et al., 2021). Therefore, PMF, ROS, and NO were measured. PMF was reduced by approximately 2% in Δ*pts*-P and Δ*pts*-P + glucose but elevated by approximately 15% in K12 + glucose compared to that in K12 (Figure 6A). These results indicate that glucose normally leads to an increase in PMF, and this effect is dependent upon the functional *pts* transport system. Interestingly, higher ROS was measured in K12 and K12 + glucose than in Δ*pts*-P and Δ*pts*-P + glucose, but exogenous glucose did not promote ROS product (Figure 6B). No difference in NO was detected among the four groups (Figure 6C). These results indicate that the absence of *pts* promoter reduces both PMF and ROS product but does not cause changes in NO. However, PMF is promoted by exogenous glucose, while ROS is not.

### Glucose-mediated PMF mechanisms

**Figure 7:**
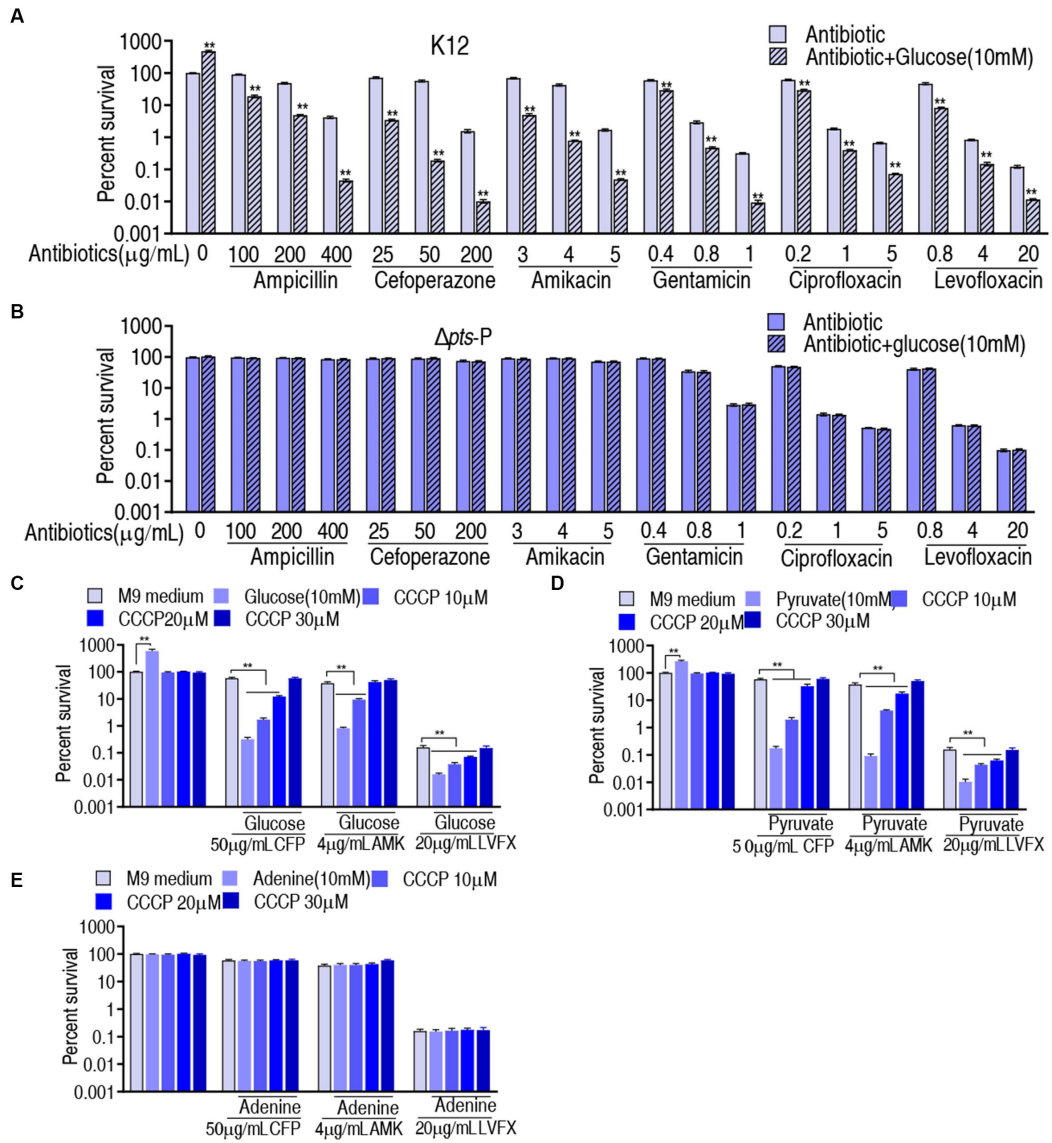
Role of PMF in glucose-mediated antibiotic resistance. **(A)** Survival of K12 in the presence or absence of glucose plus the indicated antibiotics. **(B)** Survival of Δ*pts*-P in the presence or absence of glucose plus the indicated antibiotics. **(C)** Survival of K12 in the presence of the indicated dose of CCCP plus glucose and an antibiotic. **(D)** Survival of K12 in the presence of the indicated dose of CCCP plus pyruvate and antibiotic. **(E)** Survival of K12 in the presence of the indicated dose of CCCP plus adenine and antibiotic. Significant differences are identified **p < 0.01.

The above results motivated us to explore whether the *pts*-related antibiotic resistance was implemented via PMF and ROS. To test this, we showed that exogenous glucose potentiated β-lactams (ampicillin and cefoperazone), aminoglycosides (amikacin and gentamicin), and quinolones (ciprofloxacin and levofloxacin) to kill K12 but not Δ*pts*-P (Figures 7A,B). On the one hand, PMF-related killing was demonstrated using PMF inhibitor carbonyl cyanide 3-chlorophenylhydrazone (CCCP). On the other hand, to validate that glucose fluxes the P cycle in potentiating antibiotic killing, not only glucose but also pyruvate (an upper metabolite of the P cycle) and adenine (a metabolite of the feeder stream of the P cycle) were used in this analysis. Survival of K12 was elevated with the increasing CCCP dose in the presence of cefoperazone, amikacin, or levofloxacin (Figure 7C). Similar results were obtained in the replacement of glucose with pyruvate but not with adenine (Figures 7D,E). These results indicate that PMF plays a role in the glucose-mediated regulation of antibiotic susceptibility/resistance.

### ROS-mediated killing

**Figure 8:**
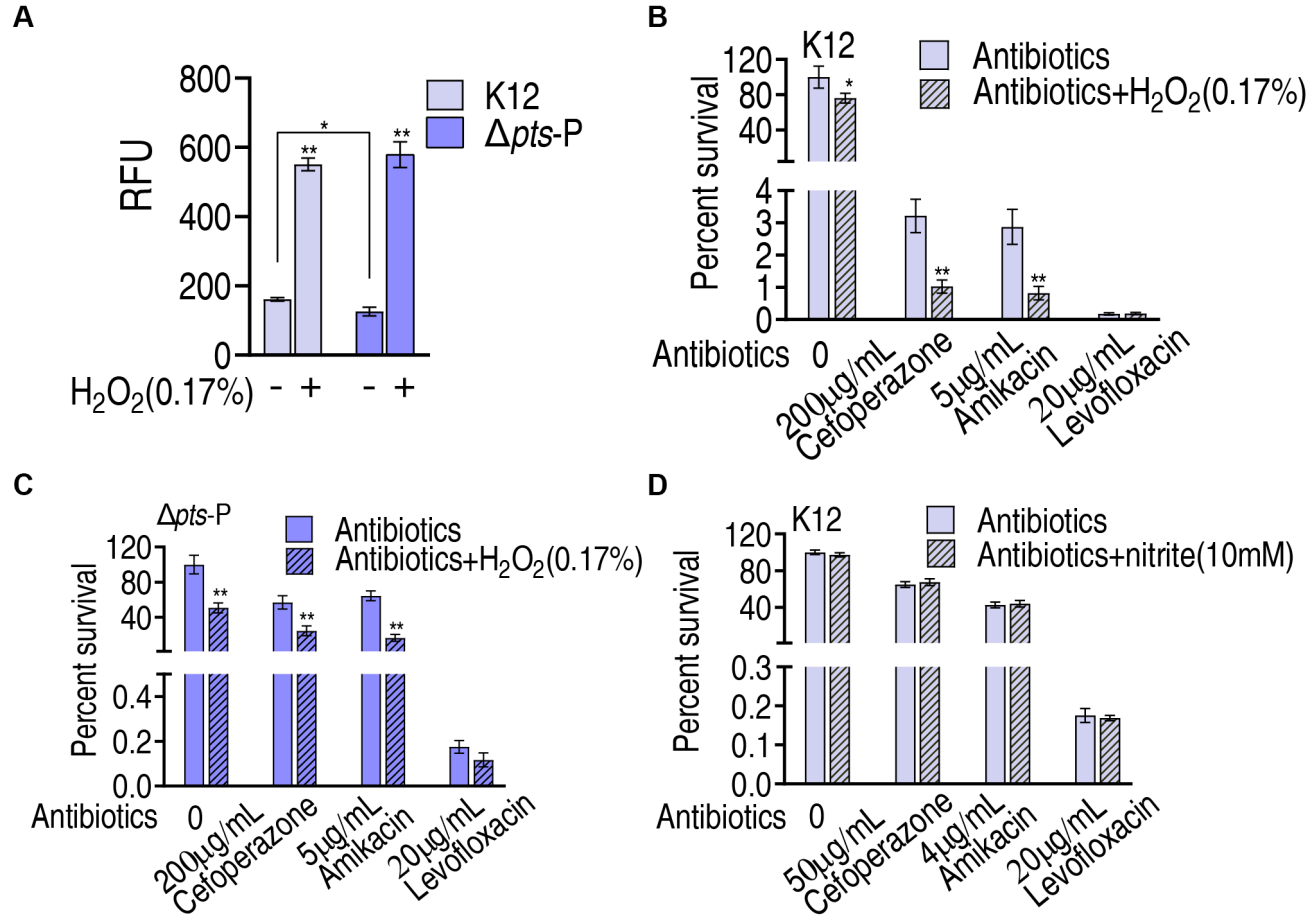
Role of ROS in glucose-mediated antibiotic resistance. **(A)** ROS measurement in K12 and Δ*pts*-P with and without 0.17% H_2_O_2_. **(B)** Survival of K12 in the presence of H_2_O_2_ plus antibiotics. **(C)** Survival of Δ*pts*-P in the presence of H_2_O_2_ plus antibiotics. **(D)** Survival of K12 in the presence or absence of nitrite plus antibiotics. Results are displayed as mean ± SEM and three biological repeats are performed. Significant differences are identified *p < 0.05, **p < 0.01.

Then, the role of ROS was explored. The findings that exogenous glucose did not increase ROS level, while higher ROS was measured in K12 than in Δ*pts*-P suggest that the loss of *pts* promoter caused ROS depression. However, whether ROS plays a role in potentiating antibacterial killing remains to be investigated. To test this, K12 and Δ*pts*-P were incubated with 0.17% H_2_O_2,_ which provides ROS. This caused the ROS level to increase approximately 3-fold in both strains (Figure 8A). Then, K12 and Δ*pts*-P were incubated with cefoperazone, amikacin, or levofloxacin in the presence or absence of 0.17% H_2_O_2_. H_2_O_2_ potentiated cefoperazone and amikacin to kill K12 and Δ*pts*-P, but no synergy with the levofloxacin was detected in both strains (Figures 8B,C). These results suggest that the depressed ROS is related to cefoperazone and amikacin resistance in Δ*pts*-P. In addition, nitrite, which provides NO, did not promote the killing efficacy of the three drugs (Figure 8D).

## Discussion

Glucose-mediated antibiotic resistance has been documented, but the underlying mechanisms are largely unknown. The II^Glc^ phosphoenolpyruvate: sugar phosphotransferase system (PTS) contains the genes encoding enzyme I (*ptsI*), HPr (*ptsH*), IIA^Glc^ (*crr*), and IICB^Glc^ (*ptsG*) (Gosset, 2005). Among them, *ptsH*, *ptsI*, and *crr* share the same promoter *pts*. Thus, the absence of *pts* promoter can be an ideal model for understanding the role of glucose in antibiotic resistance. In the present study, the model is established and employed to reveal glucose-mediated resistance to β-lactams, aminoglycosides, and quinolones. This leads to the identification of PMF and ROS as effectors for the glucose-mediated resistance to β-lactams and aminoglycosides. To quinolones, however, PMF-mediated killing is achieved only when exogenous glucose is added, suggesting that a high concentration of glucose is required for the killing via PMF. Together, glucose level confounds antibiotic sensitivity/resistance in bacteria.

PTS controls glucose transport to manage glucose metabolism (Alva et al., 2020). To explore which metabolic pathways and metabolites are influenced due to the absence of *pts*, a GC–MS-based metabolomics approach is employed to profile the metabolic state in Δ*pts*-P compared with that in K12. Meanwhile, Δ*pts*-P and K12 with glucose are used as negative and positive controls, respectively. Compared with those in K12, 15 metabolites exhibit opposite changes at abundance between K12 + glucose and Δ*pts*-P with and without glucose (2 upregulation and 13 downregulation in Δ*pts*-P with and without glucose and vice versa in K12 + glucose). These metabolites lead to the enrichment of 9 metabolic pathways. Among them, the inactivated P cycle is identified as the most affected pathway, in which malate, citric acid, and fumaric acid out of the 13 downregulated metabolites are included in Δ*pts*-P with and without glucose. Therefore, the P cycle as the most affected metabolic pathway is documented due to the loss of *pts* promoter. Notably, not all metabolites in the P cycle are reduced, for example, upregulated succinic acid, which may be because the activity of enzymes of the P cycle is not changed in proportion or because the other metabolic pathways that generate succinic acid are activated, such as glyoxylate shunt (Bhusal et al., 2017; Zhong et al., 2020).

The P cycle provides NADH for the respiratory chain, and NADH is linked to PMF generation (Su et al., 2015; Yang et al., 2018; Shimizu and Matsuoka, 2019; Jiang et al., 2020; Fan et al., 2023). Thus, the role of PMF in glucose-mediated resistance is investigated. It is shown that higher PMF is measured in K12 than in Δ*pts*-P, indicating that glucose influences PMF, which is further supported by the event that PMF is higher in K12 with exogenous glucose than without. The glucose-enabled killing by antibacterials is inhibited by CCCP in a dose-dependent manner, indicating that the glucose-mediated PMF mechanism plays a key role in the killing. Importantly, PMF-dependent killing is documented in aminoglycosides (Zhao et al., 2022), but whether the mechanism exists in β-lactams and quinolones is not defined. Our results reveal that PMF-dependent killing also plays a role in the two classes of antibacterials.

Interestingly, ROS is lower in Δ*pts*-P than K12, which is independent on exogenous glucose. Thus, glucose is not an indispensable source of ROS product. However, the loss of *pts* promoter causes ROS reduction by approximately one-third. This is because glucose nagatively regulates the cAMP/CRP system, and the reduction of cAMP or absence of *crp* correlates with the suppression of stressor-mediated ROS accumulation (Donovan et al., 2013; Colton et al., 2015; Molina-Quiroz et al., 2018; Jiang et al., 2023). Furthermore, *ptsI* deficiency lowers phenol- and ciprofloxacin-stimulated intracellular ROS levels, which is related to the PtsI-CyaA-Crp–mediated death process (Zeng et al., 2022). Thus, the dramatic reduction in ROS is attributed to a glucose-mediated indirect effect, possibly being attributed to the cAMP/CRP system. The relationship between ROS and antibiotic killing is reported, indicating that boosting ROS product is a mechanism by which antibacterials kill bacteria (Kohanski et al., 2007; Brynildsen et al., 2013; Zhang et al., 2020). However, whether the ROS reduction affects antibacterial killing as a form of antibiotic resistance is not defined. The present study indicates that the decreased ROS caused by the loss of *pts* promoter is related to resistance to β-lactams and aminoglycosides, while the elevated glucose promotes the killing efficacy caused by the three classes of antibacterials, including quinolones. These findings support the conclusion that the depressed ROS is related to the insensitivity to β-lactams and aminoglycosides and a high dose of glucose contributes to the killing efficacy of β-lactams, aminoglycosides, and quinolones.

In summary, the present study establishes a model with the absence of *pts* promoter to understand glucose-mediated antibiotic resistance mechanisms. The depressed glucose inactivates the P cycle and thereby reduces the PMF level to play a role in antibiotic resistance. Meanwhile, it depresses ROS product, which is related to insensitivity to antibiotics. These findings clarify the glucose-mediated PMF and ROS mechanisms that cause bacterial resistance/sensitivity to antibacterials.

## Materials and methods

### Bacterial strains and culture conditions

*Escherichia coli* K12 and *E. coli* K12 *pts* promoter-deleted strains were preserved in our laboratory. These strains were grown at 37°C, at 200 rpm in 50 mL LB (1% bacterial peptone, 0.5% yeast extract, and 1% NaCl) overnight and collected by centrifugation at 8000 × rpm for 3 min.

### Measurement of MIC

MIC was determined using antimicrobial susceptibility testing as previously described (Tao et al., 2022). Briefly, bacteria cultured overnight were diluted 1:100 in fresh LB medium and incubated at 37°C at 200 rpm until an optical density at 600 nm of 0.5. Subsequently, 10 μL of bacteria containing 5 × 10^4^ colony-forming units (CFU) were added to each well of a 96-well microtiter polystyrene tray, along with 100 μL of LB medium containing serial 2-fold dilutions of the antibiotic. The trays were then incubated at 37°C for 16 h. The MIC was defined as the lowest concentration of the antibiotic that visibly inhibited bacterial growth.

### Measurement of MKC

MKC was conducted following a previously described method (Zhao et al., 2021). Briefly, bacteria cultured overnight in LB medium were harvested and washed three times with saline solution before being resuspended in M9 medium to achieve an optical density at 600 nm of 0.2. Subsequently, 100 μL aliquots containing 1 × 10^7^ CFU were dispensed into individual wells of a 96-well microtiter polystyrene tray, along with 100 μL of M9 medium containing a range of antibiotic concentrations with 2-fold dilutions. The mixtures were incubated at 37°C for 8 h. The minimum killing concentration (MKC) was determined as the lowest antibiotic concentration at which visible bacterial growth inhibition occurred.

### Construction for *E. coli* K12 *pts* promoter–deleted mutants

*pst* promoter deletion strain was constructed with one-step inactivation of chromosomal genes as described previously (Jiang et al., 2020). The kanamycin-resistance cassette was amplified using pKD13 using a pair of primer *pst*-P-1F and *pst*-P-1R (*pst*-P-1F: atcgggtgagcgttatttaagcaccgcattgtttgccgatctcttcaat g ATTCCGGGGATCCGTCGACC, *pst*-P-1R: tgtgcagaccgttcggagcggtaatggtaacttcttgc tggaacattgtaTGTAGGCTGGAGCTGCTTCG. The lowercase letters are the *pst* promoter homologous sequence; the capital letters are the kanamycin gene homologous sequence). The amplified DNA was then electroporated into *E. coli* K12 cells containing pSIM5, which expresses the lambda red genes from a high-temperature-inducible promoter. Recombinants were selected on LB agar plates supplemented with kanamycin (50 μg/mL) and validated by PCR amplification of the kanamycin gene using a pair of primers, *pst*-P-2F and *pst*-P-2R (*pst*-P-2F: CACCAATGAAGAAGCGATTTCTACCG; *pst*-P-2R: CGG AGATAGTCACAACGGTACCTT), which was homologous to upstream and downstream of *pts* promoter. The *pst* promoter deletion mutants were subsequently transformed with pCP20 and cultured at 30°C then 43°C to eliminate kanamycin and chloramphenicol resistances.

### Analysis of metabolomic data

GC–MS analysis was conducted following a previously described method (Kuang et al., 2021). Briefly, 10 mL bacteria at OD_600_ = 1.0 was quenched by 1 mL pre-cooled methanol (Sigma-Aldrich). After ultrasonication and centrifugation, 1,000 μL aliquot of the supernatant was transferred into a 1.5-mL microtube, added to 10 μL ribitol (0.1 mg/mL, Sigma-Aldrich) as an internal standard, and then dried using a vacuum centrifugation device (LABCON-CO). GC–MS analysis was performed using a two-stage technique. The mass fragmentation spectra were analyzed using Xcalibur software (Thermo Fisher Scientific, version 2.1) for compound identification, utilizing the National Institute of Standards and Technology (NIST) library and NIST MS search 2.0 program. The peak areas of all identified metabolites were normalized by total mass correction. Each sample was analyzed with four biological replicates and two technical replicates.

### Membrane potential measurement

Measurement of membrane potential was performed using the BacLight Bacterial Membrane Potential Kit (Invitrogen). Then, 1 mL bacteria with a concentration of 10^6^ CFU/mL was added to 10 μL of 3 mM 3,3′-Diethyloxacarbocyanine iodide (DiOC_2_) and incubated for 30 min at 37°C 200 rpm in the dark. DiOC2 naturally emits green fluorescence. However, as the membrane voltage increases, the dye molecules accumulate on the membrane surface, undergo a change in optical properties, and emit red fluorescence. Exploiting this phenomenon, membrane voltage changes are quantified to correspond to the respective proportions. The samples were then analyzed using a FACSCalibur flow cytometer (Becton Dickinson, San Jose, CA, United States) by measuring the red light fluorescence intensity (Y mean) and green light fluorescence intensity (X mean). The membrane potential was quantified and normalized based on the intensity ratio of red fluorescence and green fluorescence. Experiments were repeated at least in three independent biological replicates.

### Measurement of enzyme activity

Enzyme activity was determined following previously described protocols (Yang et al., 2018). In brief, after bacteria were adjusted to an OD_600_ of 1.0 in 1× PBS (pH 7.0), 30 mL cells were centrifuged and then resuspended with 1 mL of 1× PBS. Then, the cells were subjected to sonication for 10 min on ice (total power 200 W, 35% output, pulse for 2 s, pause for 3 s). After centrifugation at 12,000 rpm for 10 min at 4°C, supernatants were collected, and protein concentration was determined using the BCA protein concentration determination kit (Beyotime). Subsequently, 200 μg of proteins were used for enzyme activity measurements. For PDH and KGDH measurements, the reaction mixture contained 0.5 mM MTT, 2.5 mM MgCl2, 0.5 mM PMS, 0.2 mM TPP, 2 mM sodium pyruvate/α-ketoglutarate potassium salt, and 50 mM PBS, with distilled deionized water added to a final volume of 200 μL in a 96-well plate. For SDH and MDH measurements, the reaction mixture included 0.5 mM MTT, 2.5 mM MgCl2, 0.5 mM PMS, 2 mM sodium succinate/sodium malate, and 50 mM PBS, with distilled deionized water added to 200 μL. The reaction mixtures were incubated at 37°C for 5 min and 15 min in the measurement of PDH, SDH and MDH, KGDH, respectively. Finally, the absorbance at 562 nm was measured. Experiments were repeated in three independent biological replicates.

### Antibiotic bactericidal assay by plate counting

Antibiotic bactericidal assay was conducted following established protocols (Zhao et al., 2021). The overnight bacteria were washed three times with saline solution, resuspended in M9 minimal medium supplemented with 10 mM acetate, 2 mM MgSO_4_, and 100 μM CaCl_2_, and then diluted to an OD_600_ of 0.2. The cultures were incubated in the presence or absence of glucose and/or antibiotics for 6 h at 37°C 200 rpm. To determine bacterial counts at specific time points, 100 μL aliquots of the samples were serially diluted. A volume of 5 μL from each dilution was plated onto LB agar plates and incubated at 37°C for 12 h. Survival percentage was calculated by dividing the CFU obtained from the treated sample by the CFU obtained from the untreated sample.

### Quantification of ROS production

The centrifuged bacteria were washed three times with saline solution, resuspended in M9 minimal medium, and then diluted to an OD_600_ of 0.2. After incubation with or without glucose for 6 h at 37°C 200 rpm, the cells were centrifuged, washed, and adjusted to OD600 = 0.2. A 1 mL culture of 1× 10^7^ CFU was added to 2′,7′-dichlorofluorescin diacetate (Sigma) (its final concentration was 10 μM) and incubated in the dark at 37°C for 30 min. The chemical reacted with the intracellular source of ROS, including O_2_-, H_2_O_2_, and OH-. The fluorescence of samples was measured at excitation and emission wavelengths of 485 nm and 535 nm using a Victor X5 multimode plate reader, respectively.

### Detection of nitric oxide

The determination of NO was performed using a previously described method (Kuang et al., 2021). Briefly, bacteria were cultured in M9 medium with or without glucose for 6 h at 37°C 200 rpm. The cells were then collected and washed three times with saline. After adjustment to an OD600 nm of 1.0, a 30 mL aliquot of cells was centrifuged, resuspended in 600 μL of saline, and then subjected to sonic oscillation for 7 min (total power of 200 W with 35% output, 2 s pulse, 3 s pause) over ice. Following centrifugation at 12,000 rpm for 10 min at 4°C, supernatants were collected, and protein concentration was determined using the BCA protein concentration determination kit (Beyotime, P0009). Next, 500 μL of 4 mg/mL proteins were mixed with 400 μL of buffer I and incubated at 37°C for 60 min. The reaction solution was then mixed with 300 μL of buffer II and vortexed for 30 s. After 40 min at 25°C, the supernatant was obtained by centrifugation at 1,600 rpm for 10 min. An 800 μL aliquot of the supernatant was mixed with 600 μL of color developer. After 10 min at 25°C, the absorbance was measured at 550 nm using a cuvette with a 0.5 cm optical path. OD value of blank control (same volume of protein buffer) and standard sample (0.1 mmol/L) were tested, respectively. The NO concentration (mmol/g) was calculated using the following formula: NO concentration = (Experiment group OD value – blank OD value)/(standard sample OD value - blank OD value) × standard sample concentration/protein concentration.

## Data availability statement

The original contributions presented in the study are included in the article, further inquiries can be directed to the corresponding authors.

## Author contributions

J-jT: Formal analysis, Investigation, Methodology, Writing – original draft. S-hL: Formal analysis, Investigation, Methodology, Writing – original draft. J-hW: Investigation, Writing – original draft. X-xP: Conceptualization, Writing – original draft, Writing – review & editing. HL: Conceptualization, Writing – original draft, Writing – review & editing.
